# Severe Hypothermia Induces Ferroptosis in Cerebral Cortical Nerve Cells

**DOI:** 10.3390/ijms25158086

**Published:** 2024-07-25

**Authors:** Chao-Long Lu, Jing-Jing Sha, Ru-Fei Ma, Xue-Tong Dong, Xiao-Rui Su, Bin Cong, Song-Jun Wang

**Affiliations:** Hebei Key Laboratory of Forensic Medicine, Collaborative Innovation Center of Forensic Medical Molecular Identification, Research Unit of Digestive Tract Microecosystem Pharmacology and Toxicology, Chinese Academy of Medical Sciences, College of Forensic Medicine, Hebei Medical University, No. 361 Zhong Shan Road, Shijiazhuang 050017, China; 18301414@hebmu.edu.cn (C.-L.L.); jjshas@163.com (J.-J.S.); 18200713@hebmu.edu.cn (R.-F.M.); xuetongdong@163.com (X.-T.D.); susuxr@126.com (X.-R.S.)

**Keywords:** ferroptosis, nerve cells, hypothermia, forensic, biomarker

## Abstract

Abnormal shifts in global climate, leading to extreme weather, significantly threaten the safety of individuals involved in outdoor activities. Hypothermia-induced coma or death frequently occurs in clinical and forensic settings. Despite this, the precise mechanism of central nervous system injury due to hypothermia remains unclear, hindering the development of targeted clinical treatments and specific forensic diagnostic indicators. The GEO database was searched to identify datasets related to hypothermia. Post-bioinformatics analyses, DEGs, and ferroptosis-related DEGs (FerrDEGs) were intersected. GSEA was then conducted to elucidate the functions of the Ferr-related genes. Animal experiments conducted in this study demonstrated that hypothermia, compared to the control treatment, can induce significant alterations in iron death-related genes such as PPARG, SCD, ADIPOQ, SAT1, EGR1, and HMOX1 in cerebral cortex nerve cells. These changes lead to iron ion accumulation, lipid peroxidation, and marked expression of iron death-related proteins. The application of the iron death inhibitor Ferrostatin-1 (Fer-1) effectively modulates the expression of these genes, reduces lipid peroxidation, and improves the expression of iron death-related proteins. Severe hypothermia disrupts the metabolism of cerebral cortex nerve cells, causing significant alterations in ferroptosis-related genes. These genetic changes promote ferroptosis through multiple pathways.

## 1. Introduction

The advancement of global industrialization and rising greenhouse gas emissions have made abnormal climate change one of the most urgent global challenges of our era [1,2]. In severely cold areas and seasons, unusually cold and severe convective weather caused by climate change threatens the safety of individuals, often resulting in disability and death from central nervous system disorders. The mechanisms underlying hypothermia-induced central nervous system disorders remain unclear, leading to a lack of targeted clinical treatments and specific molecular diagnostic markers in forensic practice.

Ferroptosis, a novel form of iron-dependent regulated cell death distinct from apoptosis, necrosis, and autophagy, results from the accumulation of lipid reactive oxygen species (ROS) and lipid peroxidation [3,4,5]. It plays a crucial regulatory role in the occurrence and progression of various neurological diseases and nerve injuries, including Alzheimer’s disease [6], Parkinson’s disease [7], multiple sclerosis [8], PM2.5 exposure [9], manganese exposure [10], spinal cord injury [11], hypoxic–ischemic brain injury [12], and sepsis-related encephalopathy [13]. Ferroptosis signals may propagate to neighboring cells via the release of oxidized lipids from extracellular vesicles, inducing ferroptosis in adjacent cells [14]. In astrocytes, ferroptosis stimulation can lead to nerve cell death [15]. Ferroptosis inhibitors have shown efficacy in inhibiting excitotoxicity both in vitro and in vivo, suggesting that these drugs may prevent nerve cell death by targeting astrocytes or immune cells [5,16]. The depletion of glutathione results in GPX4 inactivation and a reduction in cellular protection against lipid peroxidation, ultimately leading to ferroptosis [17]. The loss of multifunctional antioxidants like glutathione in nerve cells results in cell death [18,19]. Lipid peroxidation compromises the fluidity and stability of cell and organelle membranes, causing rupture and death. Despite the involvement of ferroptosis in multiple tissues and energy metabolism, its mechanisms under the effects of hypothermia remain unclear, particularly with regard to whether hypothermia can induce ferroptosis in nerve cells.

In this study, hypothermia-related differentially expressed genes (DEGs) from the Gene Expression Omnibus (GEO) database were integrated with ferroptosis-related genes from FerrDb for bioinformatics analysis, to identify ferroptosis-related DEGs (FerrDEGs). These potential biomarkers were then validated and examined through in vivo experiments. The objective was to identify potential biomarkers for ferroptosis under hypothermia and provide a theoretical basis for understanding the mechanisms of cerebral cortical neuron injury induced by hypothermia.

## 2. Results

### 2.1. Identification of DEGs and FerrDEGs in Hypothermia

**Figure 1:**
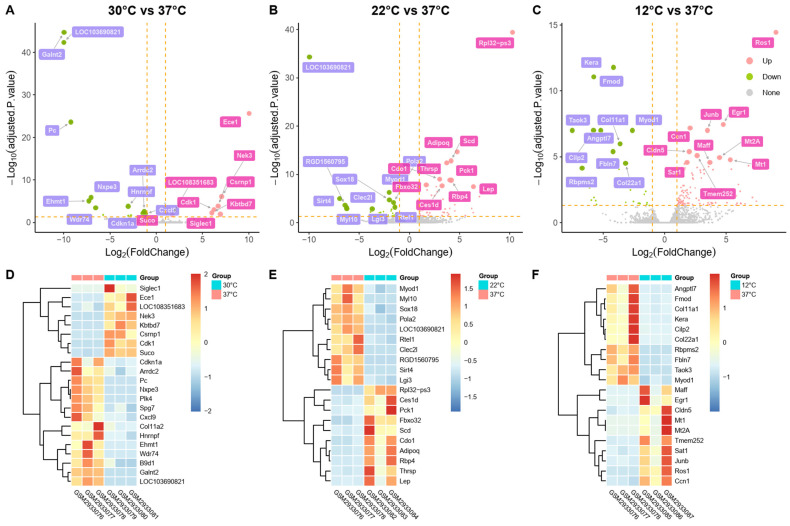
Volcano plots and heatmaps of DEGs: (**A**–**C**) Volcano plots of DEGs between treatment samples (30 °C, 22 °C, and 12 °C) and control samples (37 °C). Pink dots represent up-regulated DEGs, green dots represent down-regulated DEGs, and grey dots represent genes without significant differences. (**D**–**F**) Heatmaps of DEGs between treatment samples (30 °C, 22 °C, and 12 °C) and control samples (37 °C). Red indicates up-regulated DEGs, while blue indicates down-regulated DEGs.

After data preprocessing, DEGs from GSE109148 under hypothermia conditions were identified (Supplementary Table S1). In total, 127, 84, and 22 DEGs were identified at 30 °C, 22 °C, and 12 °C, respectively. At 30 °C, 8 genes were significantly up-regulated and 14 were significantly down-regulated (Figure 1A). At 22 °C, 108 genes were significantly up-regulated and 29 were significantly down-regulated (Figure 1B). At 12 °C, 57 genes were significantly up-regulated and 27 were significantly down-regulated (Figure 1C). Heatmaps of these DEGs show clear discrimination between the treatment and control samples, and volcano plots highlight the top 10 highly expressed and top 10 lowly expressed genes in the treatment group compared to the control group (Figure 1D–F). Ferroptosis-related genes associated with hypothermia were identified by intersecting ferroptosis-related genes with the DEGs. Five FerrDEGs were identified at 12 °C (EGR1, SAT1, SCD, FZD7, ADIPOQ) and at 22 °C (ADIPOQ, SCD, SAT1, PPARG, EGR1), all of which were up-regulated under hypothermia (Supplementary Table S2). No FerrDEGs were found at 30 °C.

### 2.2. Functional Enrichment Analyses of FerrDEGs

**Figure 2:**
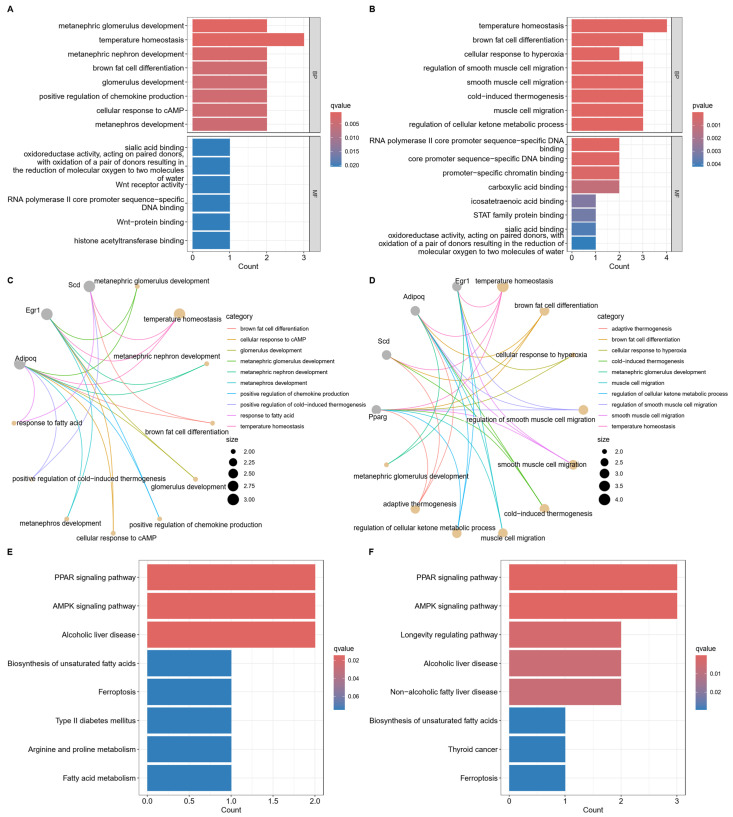
Gene Ontology (GO) and Kyoto Encyclopedia of Genes and Genomes (KEGG) enrichment analyses of FerrDEGs. (**A**) Bar plot of GO enrichment analysis of FerrDEGs at 12 °C under hypothermia. (**B**) Bar plot of GO enrichment analysis of FerrDEGs at 22 °C under hypothermia. (**C**) Cnet plot of GO enrichment analysis of FerrDEGs at 12 °C under hypothermia. (**D**) Cnet plot of GO enrichment analysis of FerrDEGs at 22 °C under hypothermia. (**E**) Bar plot of KEGG enrichment analysis of FerrDEGs at 12 °C under hypothermia. (**F**) Bar plot of KEGG enrichment analysis of FerrDEGs at 22 °C under hypothermia. BP: biological process; MF: molecular function.

To investigate the biological functions and pathways of FerrDEGs at 12 °C and 22 °C under hypothermia, GO and KEGG enrichment analyses were performed. GO enrichment analysis of FerrDEGs at 12 °C revealed significant associations with temperature homeostasis, brown fat cell differentiation, response to fatty acid, and positive regulation of cold-induced thermogenesis (Figure 2A,C and Supplementary Tables S3 and S4). Similarly, GO analysis of FerrDEGs at 22 °C indicated enrichment of temperature homeostasis, brown fat cell differentiation, and cold-induced thermogenesis (Figure 2B,D). KEGG results demonstrated that FerrDEGs at both 12 °C and 22 °C were significantly enriched in the PPAR signaling pathway, AMPK signaling pathway, biosynthesis of unsaturated fatty acids, and ferroptosis (Figure 2E,F).

### 2.3. GSEA Results

**Figure 3:**
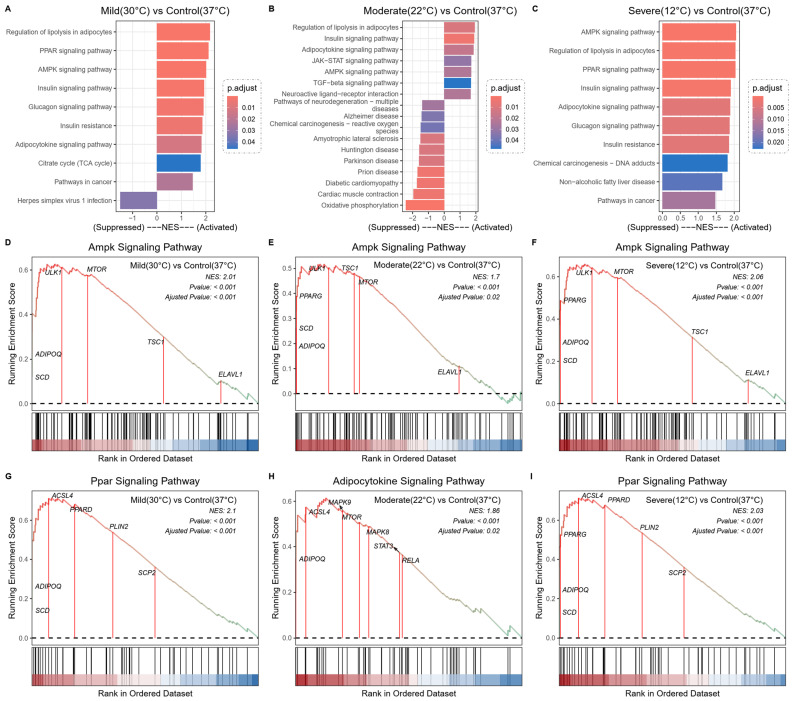
GSEA was performed to analyze the signaling pathway enrichment in different groups. (**A**–**C**) GSEA bar plots at 30 °C, 22 °C, and 12 °C. (**D**–**F**) GSEA enrichment plots showing significant enrichment of the AMPK signaling pathway at 30 °C, 22 °C, and 12 °C. (**G**–**I**) GSEA enrichment plots showing significant enrichment of the PPAR signaling pathway at 30 °C and 12 °C, and the adipocytokine signaling pathway at 22 °C.

To explore the potential pathways and gene functions associated with hypothermia, expression data were analyzed using GSEA. The GSEA results indicate that Ferr-related genes are primarily involved in the regulation of lipolysis in adipocytes, the AMPK signaling pathway, the PPAR signaling pathway, the insulin signaling pathway, and the adipocytokine signaling pathway. These pathways are shown to be activated in the bar plots (Figure 3A–C). However, some pathways were suppressed at 22 °C (Figure 3B). Notably, Ferr-related genes such as ADIPOQ, PPARG, SCD, and ACSL4 are prominently featured in the leading-edge subsets in the GSEA results (Figure 3D–I, Supplementary Table S5). The GSEA results are consistent with those of the GO and KEGG analyses based on FerrDEGs.

### 2.4. Hypothermia Induces Ultrastructural Changes in Cerebral Cortical Nerve Cells

**Figure 4:**
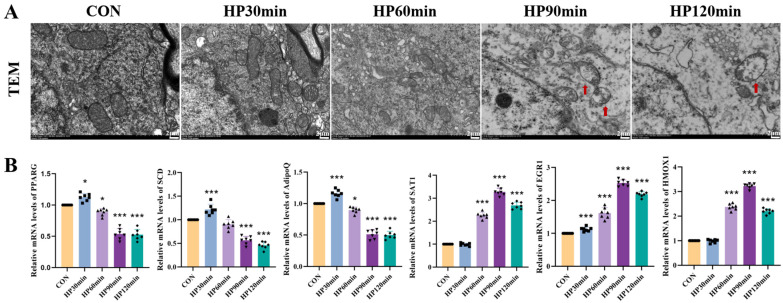
Hypothermia-induced neuronal damage and ferroptosis-related mRNA expression. (**A**) Representative TEM images of neuron nuclei in cerebral cortex tissue of mice. Scale bars = 2 μm. Hypothermia-induced mitochondrial swelling and cristae disappearance are indicated by red arrows. (**B**) Relative mRNA levels of PPARG, SCD, ADIPOQ, SAT1, EGR1, and HMOX1 in cerebral cortex tissue of mice. Data are presented as mean ± SEM; * *p* < 0.05, *** *p* < 0.001 vs. control group (*n* = 7).

Morphological changes in mitochondria are the most representative feature of ferroptosis. We used TEM images to observe the mitochondria of cerebral cortical nerve cells. The results showed a decrease or complete disappearance of mitochondrial cristae in nerve cells; condensation of the mitochondrial membrane; and rupture and shrinkage of the outer mitochondrial membrane in the HP90 min and HP120 min groups. These changes were not accompanied by typical apoptotic features such as cell shrinkage, chromatin condensation, or apoptotic body formation. The observed manifestations are consistent with the ultrastructural characteristics of ferroptosis [20,21] (Figure 4A).

### 2.5. Real-Time Quantitative PCR

The results demonstrate that as hypothermia duration increased, the core body temperatures of the mice progressively decreased. In the cerebral cortex tissue, the expression of PPARG, SCD, and ADIPOQ showed a significant downward trend compared to the control group. Conversely, the levels of SAT1, EGR1, and HMOX1 significantly increased (Figure 4B).

### 2.6. Severe Hypothermia Induces Ferroptosis-Related Protein Expression and Lipid Peroxidation in the Cerebral Cortex

#### 2.6.1. Hypothermia Induces Iron Accumulation in Cerebral Cortex Tissue

**Figure 5:**
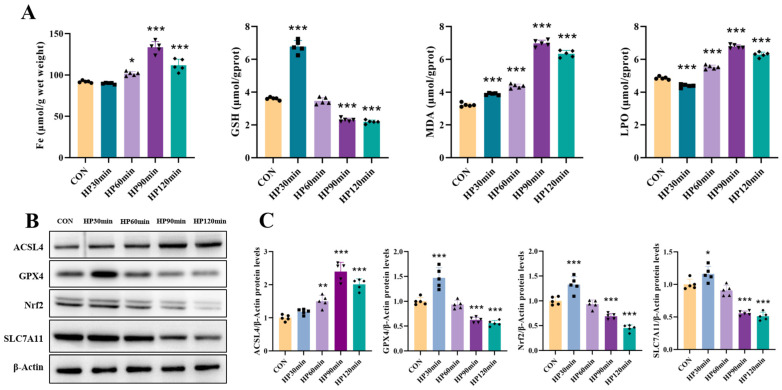
Hypothermia-induced lipid peroxidation and expression of ferroptosis-related proteins. (**A**) Levels of GSH, MDA, LPO, and iron content in cerebral cortex tissue of mice. (**B**) Western blotting analysis of ACSL4, GPX4, NRF2, and SLC7A11 expression in cerebral cortex tissue. (**C**) Quantification of protein levels shown in (**B**). Data are presented as mean ± SEM; * *p* < 0.05, ** *p* < 0.01, *** *p* < 0.001 vs. control group (*n* = 5).

Iron ion accumulation can lead to tissue damage, and iron metabolism disorders are essential for lipid peroxide accumulation and the activation of the ferroptosis pathway [22]. Our findings demonstrate that as their core body temperatures decreased, iron ion content in the cerebral cortex tissue of the mice significantly increased compared to that of the control group. This suggests that a reduction in core body temperature induces iron ion accumulation in the cerebral cortex tissue (Figure 5A).

#### 2.6.2. Severe Hypothermia Increases Lipid Peroxides in Cerebral Cortex Tissue

Lipid peroxide deposition and oxidative stress are critical steps in the development of ferroptosis [23,24]. Our results indicate that as their core body temperatures decreased, hypothermia significantly increased MDA and LPO levels in the cerebral cortex tissue of the affected mice compared to the control group. Conversely, their GSH levels progressively declined with their decreasing core body temperatures. These findings suggest that hypothermia reduces antioxidant levels and promotes lipid peroxide deposition in the cerebral cortex tissue (Figure 5A).

#### 2.6.3. Severe Hypothermia Induces Ferroptosis-Related Protein Expression in the Cerebral Cortex

SLC7A11 is a crucial component of the glutamate/cystine transporter at the cell membrane, facilitating cystine uptake and glutathione biosynthesis to protect cells from ferroptosis. Glutathione peroxidase 4 (GPX4), an antioxidant enzyme, detects oxidative stress and converts lipid hydroperoxides into non-toxic lipid alcohols to prevent ferroptosis. Both SLC7A11 and GPX4 are central regulators of ferroptosis. Acyl-CoA synthetase long-chain family member 4 (ACSL4) is a key enzyme in polyunsaturated fatty acid (PUFA) metabolism, influencing cell sensitivity to ferroptosis. Studies indicate that a moderate decline in core body temperature enhances the brain tissue’s anti-ferroptosis capability. However, in our study, as the core body temperatures of the mice continued to fall, the expression of NRF2, SLC7A11, and GPX4 in the cerebral cortex tissue significantly decreased, while ACSL4 expression significantly increased compared to that of the control group. Overexpression of ACSL4 heightens cell sensitivity to ferroptosis, while decreased levels of SLC7A11 and GPX4 are markers of ferroptosis [15,25,26]. These findings suggest that ferroptosis is involved in nerve cell injury caused by severe hypothermia (Figure 5B,C).

### 2.7. Ferroptosis Inhibitors Mitigate Ferroptosis in the Cerebral Cortex Induced by Hypothermia

#### 2.7.1. Fer-1 Effectively Mitigates the Core Body Temperature Drop Induced by a Low-Temperature Environment

**Figure 6:**
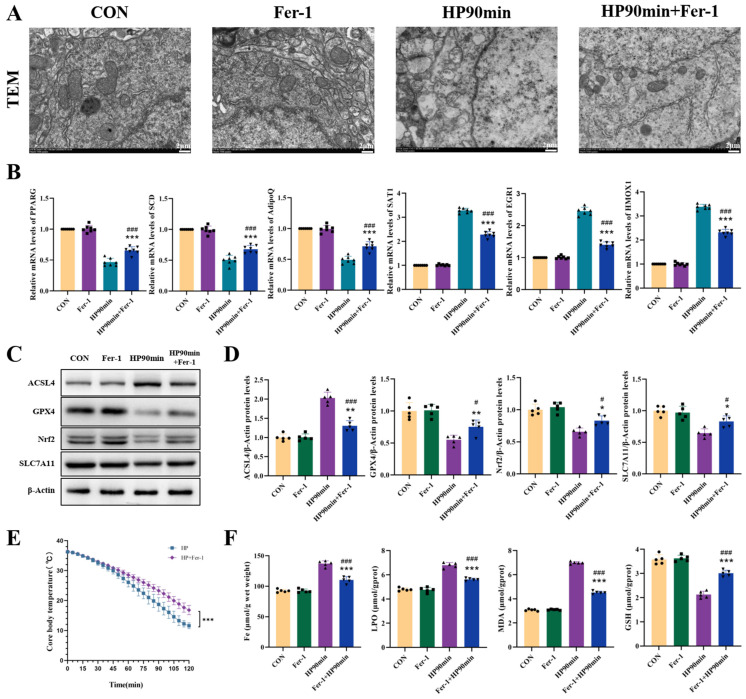
Fer-1 alleviates hypothermia-induced neuronal damage and mitigates the decreased expression of ferroptosis-related proteins. (**A**) The representative TEM images of neuron nuclei in the cerebral cortex. Scale bars = 2 μm. Fer-1 alleviated hypothermia-induced mitochondrial swelling and the disappearance of cristae. (**B**) The relative mRNA levels of PPARG, SCD, ADIPOQ, SAT1, EGR1, and HMOX1 in the cerebral cortex tissue of mice (*n* = 7). (**C**) The expression of ACSL4, GPX4, NRF2, and SLC7A11 in the cerebral cortex, assessed by western blotting analysis. (**D**) Quantification of the protein levels shown in (**C**). (**E**) As the time spent in the 2–6 °C environment increased, the core body temperatures of the mice gradually decreased. (**F**) The levels of LPO, MDA, GSH, and iron content in the cerebral cortex tissue of mice. The data are presented as mean ± SEM; * *p* < 0.05, ** *p* < 0.01, *** *p* < 0.001 vs. the control group (*n* = 5). # *p* < 0.05, ### *p* < 0.001 vs. the HP90 min group (*n* = 5).

The core body temperature of a mouse significantly influences its mental state under hypothermia, with lower temperatures correlating with worse mental consciousness. This study discovered that Fer-1 slows down the decrease in core body temperature of mice and improves their mental state (Figure 6E).

#### 2.7.2. Fer-1 Alleviates Hypothermia-Induced Mitochondrial Ultrastructural Damage in Neurons

Previous studies have found that hypothermia causes mitochondrial swelling and the disappearance of cristae in cerebral cortical neurons, which are changes characteristic of ferroptosis. To further confirm whether hypothermia causes ferroptosis, the ferroptosis inhibitor Fer-1 was employed in this study. The results show that Fer-1 alleviated hypothermia-induced mitochondrial swelling and the disappearance of cristae in cerebral cortex neurons (Figure 6A).

#### 2.7.3. Fer-1 Enhances Anti-Ferroptosis-Related Gene Expression

Previous studies have identified significant expression of ferroptosis-related genes in hypothermic cerebral cortex tissue, triggering ferroptosis. To further verify the mechanism of hypothermia-induced ferroptosis in cerebral cortex tissue, the ferroptosis inhibitor Fer-1 was employed in this study. The results show that Fer-1 increased the expression of PPARG, SCD, and ADIPOQ while reducing the expression of SAT1, EGR1, and HMOX1 in hypothermic cerebral cortex tissue. These findings indicate that the ferroptosis-related genes PPARG, SCD, ADIPOQ, SAT1, EGR1, and HMOX1 play a role in the ferroptosis process induced by hypothermia in cerebral cortex tissue (Figure 6B).

#### 2.7.4. Fer-1 Attenuates Hypothermia-Induced Iron and Lipid Peroxide Deposition and Enhances Resistance to Oxidative Damage

The deposition of iron ions and lipid peroxides, along with the depletion of antioxidants, is central to ferroptosis. This study found that Fer-1 effectively reduces the hypothermia-induced accumulation of iron ions and lipid peroxides (MDA and LPO) in the cerebral cortex tissue while increasing the antioxidant GSH content. These findings demonstrate that the ferroptosis inhibitor Fer-1 can effectively mitigate ferroptosis in the cerebral cortex that has been induced by hypothermia (Figure 6F).

#### 2.7.5. Fer-1 Enhances Anti-Ferroptosis-Related Protein Expression in the Cerebral Cortex

To further elucidate the mechanism of hypothermia-induced ferroptosis in cerebral cortex tissue, Fer-1 was found to enhance the expression of antioxidant substances NRF2, SLC7A11, and GPX4 while down-regulating ACSL4 to increase resistance to ferroptosis. This indicates that the NRF2/SLC7A11/GPX4/ACSL4 pathway is involved in the process of hypothermia-induced ferroptosis in cerebral cortex nerve cells (Figure 6C,D).

## 3. Discussion

Ferroptosis is a type of programmed cell death induced by lipid peroxidation through an iron-dependent pathway, characterized by unique morphological and biological features [3,27]. It involves increased ROS, iron-dependent lipid peroxidation, and plasma membrane damage, all driven by metabolic dysfunction [5,28]. This study found that prolonged hypothermia resulted in a gradual decrease in core body temperature, transitioning mice from motor excitation to motor inhibition and coma. Concurrently, iron ions accumulated, lipid peroxidation markers such as glutathione (GSH) decreased, and lipid peroxide (LPO) and malondialdehyde (MDA) rose. The ferroptosis-related proteins SLC7A11, NRF2, and GPX4 were significantly down-regulated, while ACSL4 was significantly up-regulated. Administration of the ferroptosis inhibitor Fer-1 effectively improved the expression of SLC7A11, NRF2, and GPX4 and reduced lipid peroxidation products. These findings suggest that metabolic dysfunction induced by hypothermia is a major factor driving ferroptosis.

Low temperature induces lipid metabolism disorders and triggers ferroptosis-related gene expression.

Peroxisome proliferator-activated receptor gamma (PPARG), a subtype of the steroid hormone receptor superfamily, acts as a fatty acid sensor and regulates various aspects of lipid metabolism, playing a key role in adipogenesis [29]. Bioinformatics analysis revealed a close association between PPARG and hypothermia-induced ferroptosis. This study observed that with a gradual decline in core body temperature, PPARG levels significantly decreased, alongside a notable down-regulation of SLC7A11 and NRF2. Administration of Fer-1 significantly elevated PPARG levels and improved the expression of SLC7A11 and NRF2 under hypothermia. Previous studies have shown that PPARG knockdown mitigates erastin-induced reductions in SLC7A11 and NRF2, maintaining cellular redox homeostasis and reducing ferroptosis [30,31]. These findings indicate that hypothermia disrupts neuronal lipid metabolism, leading to a significant decrease in PPARG and promoting ferroptosis.

Sterol CoA desaturase (SCD) is a lipid-modifying enzyme that catalyzes the conversion of saturated fatty acids into monounsaturated fatty acids [32]. SCD protects cells from ferroptosis by altering lipid membrane composition [33]. Bioinformatic analysis revealed a close relationship between SCD and hypothermia-induced ferroptosis. Experimentally, this study found that SCD levels significantly decreased with the gradual decline in core body temperature, accompanied by a notable down-regulation of SLC7A11 and GPX4 expression. Administration of Fer-1 significantly increased hypothermia-induced SCD levels and improved the expression of NRF2 and SLC7A11. Previous studies have shown that inhibition of SCD alters membrane phospholipid composition, reducing monounsaturated fatty acyl chains and increasing polyunsaturated fatty acyl chains, thereby decreasing membrane-located antioxidants. This change in membrane composition provides more substrates for lipid oxidation, inducing ferroptosis. These findings suggest that low temperatures alter neuronal cell membrane composition and increase sensitivity to ferroptosis by affecting SCD function.

Adiponectin (ADIPOQ) is a 30 kDa lipid factor encoded by the ADIPOQ gene. In the cerebral cortex, adiponectin primarily exerts its biological function by binding to its receptor ADIPOR1 [34]. Bioinformatics analysis revealed a close association between ADIPOQ and hypothermia-induced ferroptosis. This study found that hypothermia significantly decreased ADIPOQ levels and down-regulated the expression of NRF2 and SLC7A11. Administration of Fer-1 significantly mitigated the hypothermia-induced decrease in ADIPOQ and improved the expression of SLC7A11 and NRF2. Previous studies have shown that AdipoR1 regulates the expression of SLC7A11 through NRF2 [35,36]. SLC7A11 appears to be a key downstream target, protecting cells from IR-induced ferroptosis by promoting SLC7A11 expression.

Hypothermia Impairs Polyamine Metabolism and Triggers Ferroptosis-Related Gene Expression.

SAT1 is a rate-limiting enzyme involved in polyamine catabolism, typically present at low levels. SAT1 catalyzes the acetylation of spermidine and spermine, forming N1-acetylspermidine and N1-acetylspermine, which are then either exported from cells or converted back to putrescine or spermidine by N1-acetylpolyamine oxidase [37]. Bioinformatic analysis revealed a close association between SAT1 and hypothermia-induced ferroptosis. This study found that SAT1 levels significantly increased with the gradual decline in core body temperature, accompanied by a notable down-regulation of SLC7A11 and GPX4 expression. Administration of Fer-1 significantly mitigated the hypothermia-induced increase in SAT1 and improved the expression of SLC7A11 and NRF2. Previous studies have shown that overexpression of SAT1 leads to the overall depletion of spermidine and spermine while the levels of putrescine, N1-acetylspermidine, and N1-acetylspermine increase [38]. Polyamine catabolism by SAT1 generates H_2_O_2_ and increases the presence of lipid peroxidation products [39,40,41].

Hypothermia Induces Metabolic Impairment and Triggers Ferroptosis-Related Gene Expression.

EGR1, a zinc finger transcription factor of the early gene family, is rapidly induced by hypoxia, growth factors, and other stimuli [42]. As a redox-regulated transcription factor, EGR1 is activated by high ROS concentrations [43]. Bioinformatics analysis identified a strong correlation between EGR1 and hypothermia-induced ferroptosis. During the experiment performed in this study, hypothermia significantly increased ROS and EGR1 levels, and also caused notable down-regulation of SLC7A11 and GPX4 expression. Fer-1 administration markedly reduced the ROS and EGR1 levels induced by hypothermia, while enhancing the expression of SLC7A11 and GPX4. Previous research has shown that EGR1 knockdown increases SLC7A11 and GPX4 expression, accompanied by higher GSH levels and reduced lipid peroxides. Additionally, ROS-mediated oxidative stress during myocardial I/R injury is closely linked to EGR1. Fer-1 significantly decreases EGR1 expression in I/R-induced AKI, suggesting that Fer-1 inhibits ferroptosis by suppressing EGR1 expression [44]. These findings indicate that hypothermia activates EGR1, triggering ferroptosis through neurometabolic disorders and excessive ROS production.

Heme oxygenase 1 (HO-1), encoded by the HMOX1 gene, is a rate-limiting enzyme that regulates redox reactions and converts heme into bilirubin, regulated by upstream NRF2 signaling [45]. HO-1 plays a key role in iron production, transport, and export, and is involved in ferroptosis [46]. Bioinformatics analysis revealed a strong correlation between HMOX1 and hypothermia-induced ferroptosis. This study found that hypothermia significantly increased HMOX1 levels, accompanied by substantial iron ion accumulation and a notable down-regulation of SLC7A11 and GPX4 expression. Administration of Fer-1 significantly reduced hypothermia-induced HMOX1 levels and improved the expression of SLC7A11 and GPX4. Previous research has shown that continuous overexpression of HO-1 promotes iron ion production and the inactivation of iron regulatory protein-1 (IRP-1), leading to the collapse of iron transport regulatory signals, extracellular iron accumulation, and intracellular iron overload in nerve cells. Additionally, HO-1 overexpression increases hepcidin levels and leads to the passivation or degradation of the iron export protein (FPN1), blocking iron export and causing intracellular iron overload in nerve cells [47]. Lowering HO-1 can block iron’s production and transport into cells and reduce hepcidin secretion, increasing FPN1 expression and promoting the export and metabolism of overloaded intracellular iron [48]. These findings suggest that hypothermia triggers a significant increase in neural HMOX1, leading to iron ion accumulation and ferroptosis due to iron metabolism disorders.

Our study still has certain limitations, because the response of mice to hypothermia is not completely consistent with that of humans. We have not collected enough human samples yet, and can only use animals to do some relative research. We hope that our research can guide more scholars to carry out research combined with clinical practice that can guide clinical work. We also strive to closely combine research with clinical practice and translate basic research into applications.

## 4. Materials and Methods

### 4.1. Data Source

The GSE109148 dataset [49] was obtained from the Gene Expression Omnibus (GEO) database (http://www.ncbi.nlm.nih.gov/geo, accessed on 11 December 2022). This dataset includes four groups (control at 37 °C, mild at 30 °C, moderate at 22 °C, and severe at 12 °C hypothermia) consisting of three pathogen-free 9-week-old rats per group. The data were based on the GPL19052 platform (Illumina MiSeq, San Diego, CA, USA). Additionally, 221 ferroptosis-related genes were acquired from the FerrDb database v2 [50] (accessed on 21 December 2022) after removing duplicates. These genes encompass drivers, suppressors, and markers.

### 4.2. Identification of FerrDEGs

The dataset was analyzed using Grein [51]. DEGs were identified based on the cutoff criteria of an adjusted *p*-value < 0.05 and a |log2 fold-change (FC)| > 1. The DEGs were visualized using R packages (pheatmap, tidyverse, ggplot2, ggrepel, cowplot) to generate heatmaps and volcano plots. The intersections of DEGs and ferroptosis-related genes were obtained using R (version 4.2.2) and InteractiVenn [52], and these intersected genes were considered FerrDEGs.

### 4.3. Functional Enrichment Analysis for FerrDEGs

The clusterProfiler [53] package in R was utilized to perform Gene Ontology (GO) and Kyoto Encyclopedia of Genes and Genomes (KEGG) analyses on FerrDEGs with a significance threshold of *p* < 0.05. These analyses identified the significant functions and signaling pathways associated with the FerrDEGs.

### 4.4. Gene Set Enrichment Analysis (GSEA)

All genes were ranked according to the logFoldChange (logFC) values derived from the differential expression analysis. GSEA was conducted using the R packages clusterProfiler [53,54] and msigdbr (https://github.com/igordot/msigdbr (accessed on 18 July 2024)). The analyses were performed with the default parameters, including a *p*-value cutoff of 0.05.

### 4.5. Animal Experiments

All animal experiments received approval from the Laboratory Animal Management Committee of Hebei Medical University (approval number: 2022132). Ninety-six male C57/6N mice, 8 weeks old and weighing 20 ± 2 g, were obtained from Beijing Weitong Lihua Co., Ltd. (Beijing, China). The mice were maintained under specific pathogen-free (SPF) conditions, at a constant temperature of 23 ± 2 °C, with controlled humidity at 50% and a 12 h light/dark cycle.

### 4.6. Surgery and Core Temperature Monitoring

Mice were anesthetized with isoflurane (5% for induction, 2–3% for maintenance) and their abdominal hair was shaved. Following disinfection of the skin with iodophor and alcohol, a longitudinal incision was made in the mid-abdomen, and a disinfected thermometry capsule was placed into the abdominal cavity. The experiment commenced after one week of adaptive feeding.

### 4.7. Model Preparation and Sample Extraction

The experimental mice were divided into a control group (CON) and a hypothermia group (HP30 min, HP60 min, HP90 min, HP120 min). Under isoflurane gas anesthesia, the experimental mice had their whole body hair shaved, with particular attention paid to the head. They were then placed in a 2–6 °C box to simulate outdoor conditions lacking adequate warmth. The control group underwent decapitation under anesthesia, and cerebral cortex tissue was collected for experimental research. To further investigate the role of ferroptosis in severe hypothermia, the specific ferroptosis inhibitor Fer-1 (1 mg/kg) was administered one hour before placing the mice in the hypothermia box.

### 4.8. Transmission Electron Microscope (TEM)

The observation focused on the cerebral cortex, with the tissue processed into ultrathin sections (50 nm) and stained with uranium and cobalt following standard procedures. All samples were examined using a TEM (Hitachi HT7800, Tokyo, Japan).

### 4.9. RT-PCR Analysis

Cerebral cortex tissue samples (60 mg) underwent RNA extraction using 1000 µL TRIzol reagent (Thermo Scientific, Waltham, MA, USA), per the manufacturer’s instructions. Quantification of RNA employed the NanoDrop ND-1000 (NanoDrop Technologies, Wilmington, DE, USA), while RNA integrity was evaluated using the Bioanalyzer 2100 system (Agilent Technologies, Santa Clara, CA, USA). Total RNA was reverse transcribed into cDNA via the PrimerScript Reverse Transcription Kit with the gDNA Eraser (Takara Bio Inc., Kusatsu, Japan). The PrimeScript RT Reagent Kit and TB Green Premix Ex Taq II (Takara Bio Inc.) facilitated cDNA synthesis and quantitation. mRNA quantification occurred on an ABI 7500 real-time PCR system, following the manufacturer’s protocol. Ct values for each target molecule were derived by calculating the arithmetic mean from triplicate technical replicates. The 2^−ΔΔCt^ method determined mRNA expression relative to 18S rRNA. The following primer sequences were used in this study:

ADIPOQ forward primer: 5′ TATGGGGAAGGGGACAACAATG 3′;

ADIPOQ reverse primer: 5′ GTCGCCTGTTCTTTGATTCTCG 3′;

EGR1 forward primer: 5′ ACCTGACCACAGAGTCCTTTTC 3′;

EGR1 reverse primer: 5′ TTCAGGCCACAAAGTGTTGC 3′;

PPARG forward primer: 5′ TTTCAAGGGTGCCAGTTTCG 3′;

PPARG reverse primer: 5′ TGGACACCATACTTGAGCAGAG 3′;

SCD forward primer: 5′ TGGCACATCAACTTCACCAC3′;

SCD reverse primer: 5′ AAGACAGCGGCCTTAGAAAC 3′;

SAT1 forward primer: 5′ ATTACCAGTGGCGTTGTTGC 3′;

SAT1 reverse primer: 5′ GCCTCCAAACCACATACATGAC 3′;

HMOX1 forward primer: 5′ TGCCTGGCTCTCTTTTCTTG 3′;

HMOX1 reverse primer: 5′ TGCTGGTTTCCAAGTTCAGG 3′;

GAPDH forward primer: 5′ TCCCTCAAGATTGTCAGCAATG 3′;

GAPDH reverse primer: 5′ GATCCACAACGGATACATTGG 3′.

### 4.10. Western Blotting Analysis

Protein extracts were obtained from cerebral cortex tissue using a mammalian protein extraction kit (Pearce, Thermo Fisher Scientific, Rockford, IL, USA). Protein concentration was measured with a BCA protein assay kit. Under reducing conditions, 50 µg of protein extract was separated by SDS-PAGE and electrophoretically transferred to polyvinylidene fluoride (PVDF) membranes. Specific antibodies were used to detect protein expression: SLC7A11 (ab307601, Abcam, Cambridge, UK), GPX4 (ab125066, Abcam), NRF2 (80593-1-RR, Proteintech, San Diego, CA, USA), and ACSL4 (ab155282, Abcam). Subsequent incubation with horseradish peroxidase-labeled secondary antibodies and chemiluminescence detection allowed the analysis of the optical density of target bands using a gel image processing system.

### 4.11. Enzyme-Linked Immunosorbent Assay (ELISA)

Cerebral cortex tissue, stored at −80 °C, was collected in order to determine the protein levels of MDA (E-BC-K025-M, Elabscience, Houston, TX, USA), GSH (E-BC-K030-M, Elabscience), and LPO (E-BC-K176-M, Elabscience) using specific ELISA kits according to the manufacturer’s instructions.

### 4.12. Iron Determination

The iron level in the cerebral cortex tissue was measured using an iron assay kit (MAK025, Sigma-Aldrich, St. Louis, MO, USA) according to the manufacturer’s instructions. The cerebral cortex tissue was homogenized in iron assay buffer and centrifuged (16,000× *g*, 10 min, 4 °C) to remove insoluble substances. For ferrous iron measurement, 50 μL of the sample was added to a 96-well plate and the volume was adjusted to 100 μL with assay buffer. Additionally, 5 μL of iron reducer was added to each sample well to convert trivalent iron to ferrous iron, allowing for the measurement of total iron levels. The reactants were incubated in the dark at 30 °C for 25 min. Subsequently, 100 μL of iron probe was added to each well and incubated for 60 min, and absorbance was measured at 593 nm. The iron and total iron contents were calculated based on the standard curve. Furthermore, the protein level of the cerebral cortex tissue was measured using the bicinchoninic acid (BCA) kit (23227, Thermo Fisher Scientific), and the iron level was normalized to the protein concentration.

### 4.13. Statistical Analysis

Data analysis was conducted using R software (version 4.2.2). Data are presented as mean ± SEM from three experimental repeats. Statistical differences between two groups were assessed using Student’s *t*-test, while comparisons among three or more groups were evaluated using ANOVA followed by Bonferroni multiple comparisons. *p*-values of less than 0.05 indicated statistical significance.

## 5. Conclusions

Severe hypothermia causes metabolic disorders in cerebral cortex nerve cells, significantly altering ferroptosis-related genes such as PPARG, SCD, ADIPOQ, SAT1, EGR1, and HMOX1. These changes ultimately induce ferroptosis through multiple pathways. This study demonstrates that Fer-1 can alleviate hypothermia-induced ferroptosis and improve neurological function in mice. Targeted inhibition of ferroptosis may be a new method for treating central nervous system dysfunction caused by severe hypothermia. Studying ferroptosis under hypothermic conditions involves various tissues and organs and entails complex mechanisms. Future research will focus on the impact of organs, cells, gender, age, and other factors in hypothermic death under different conditions. These issues should be addressed in subsequent studies.

## Data Availability

In this study, public datasets were downloaded and analyzed, and they can be found in the GEO data repository under the following accession number: GSE109148.

## Supplementary Materials

The following supporting information can be downloaded at: https://www.mdpi.com/article/10.3390/ijms25158086/s1.
